# Efficacy and Safety of Denosumab vs Zoledronic Acid in OI Adults: A Prospective, Open-Label, Randomized Study

**DOI:** 10.1210/clinem/dgae012

**Published:** 2024-01-05

**Authors:** Xiaoyun Lin, Jing Hu, Bingna Zhou, Xiaojie Wang, Qian Zhang, Yan Jiang, Ou Wang, Weibo Xia, Xiaoping Xing, Mei Li

**Affiliations:** Department of Endocrinology, National Health Commission Key Laboratory of Endocrinology, Peking Union Medical College Hospital, Chinese Academy of Medical Sciences & Peking Union Medical College, Beijing 100730, China; Department of Endocrinology, National Health Commission Key Laboratory of Endocrinology, Peking Union Medical College Hospital, Chinese Academy of Medical Sciences & Peking Union Medical College, Beijing 100730, China; Department of Endocrinology, National Health Commission Key Laboratory of Endocrinology, Peking Union Medical College Hospital, Chinese Academy of Medical Sciences & Peking Union Medical College, Beijing 100730, China; Department of Endocrinology, National Health Commission Key Laboratory of Endocrinology, Peking Union Medical College Hospital, Chinese Academy of Medical Sciences & Peking Union Medical College, Beijing 100730, China; Department of Endocrinology, National Health Commission Key Laboratory of Endocrinology, Peking Union Medical College Hospital, Chinese Academy of Medical Sciences & Peking Union Medical College, Beijing 100730, China; Department of Endocrinology, National Health Commission Key Laboratory of Endocrinology, Peking Union Medical College Hospital, Chinese Academy of Medical Sciences & Peking Union Medical College, Beijing 100730, China; Department of Endocrinology, National Health Commission Key Laboratory of Endocrinology, Peking Union Medical College Hospital, Chinese Academy of Medical Sciences & Peking Union Medical College, Beijing 100730, China; Department of Endocrinology, National Health Commission Key Laboratory of Endocrinology, Peking Union Medical College Hospital, Chinese Academy of Medical Sciences & Peking Union Medical College, Beijing 100730, China; Department of Endocrinology, National Health Commission Key Laboratory of Endocrinology, Peking Union Medical College Hospital, Chinese Academy of Medical Sciences & Peking Union Medical College, Beijing 100730, China; Department of Endocrinology, National Health Commission Key Laboratory of Endocrinology, Peking Union Medical College Hospital, Chinese Academy of Medical Sciences & Peking Union Medical College, Beijing 100730, China

**Keywords:** osteogenesis imperfecta, adult, denosumab, zoledronic acid, bone mineral density, trabecular bone score

## Abstract

**Context:**

The comparative effectiveness of denosumab and zoledronic acid for adult patients with osteogenesis imperfecta (OI) has not been established.

**Objective:**

To evaluate the efficacy and safety of denosumab and zoledronic acid in adult patients with OI.

**Methods:**

This was a prospective, open-label study. Patients were randomized to receive denosumab 60 mg every 6 months or zoledronic acid 5 mg once for 12 months. Pathogenic mutations of OI were identified by next-generation sequencing and confirmed by Sanger sequencing. Percentage changes in the areal bone mineral density (aBMD), trabecular bone score (TBS), and bone turnover biomarkers (BTMs) from baseline to 6 and 12 months of treatment, as well as safety, were evaluated.

**Results:**

A total of 51 adults with OI (denosumab: 25, zoledronic acid: 26) were included, of whom 49 patients had identified pathogenic mutations. At 12 months, aBMD at the lumbar spine and total hip significantly increased by 4.34% (*P* = .005) and 1.45% (*P* = .023) in the denosumab group and by 4.92% (*P* = .006) and 2.02% (*P* = .016) in the zoledronic acid group, respectively. TBS showed an increasing trend by 1.39% and 2.70% in denosumab and zoledronic acid groups, respectively. Serum levels of β-isomerized carboxy-telopeptide of type I collagen and alkaline phosphatase markedly decreased after denosumab treatment. Percentage changes in aBMD, TBS, and BTMs during the treatment were similar between the 2 groups. Patients with OI with milder phenotypes showed a significantly higher increase in the TBS after 12 months of denosumab treatment than those with more severe phenotypes (*P* = .030). During the study period, the denosumab group had fewer adverse events than the zoledronic acid group.

**Conclusion:**

Denosumab effectively increases aBMD in adults with OI, with similar efficacy to zoledronic acid. Long-term and large-sample studies are needed to confirm the antifracture efficacy and safety of denosumab in adult patients with OI.

Osteogenesis imperfecta (OI) is the most common monogenic inherited skeletal dysplasia that is phenotypically and genetically heterogeneous, with an estimated incidence of 1 in 15 000 to 20 000 live neonates (1). Bone fragility, recurrent fractures, skeletal deformities, and growth retardation are the hallmarks of OI, and patients may have extraosseous manifestations, such as blue sclera, dentinogenesis imperfecta, joint hypermobility, hearing impairment, and cardiovascular complications (1). The majority of OI cases are caused by monoallelic mutations in genes encoding type I collagen, with an autosomal dominant inheritance pattern (2). The remaining patients with OI have autosomal recessive forms due to biallelic mutations in genes that encode proteins involved in collagen processing, post-translational modification, and crosslinking of type I collagen or in genes involved in osteoblast differentiation or bone mineralization (2). The clinical spectrum of OI ranges widely from mild to lethal, including the mildest form (type I), perinatal lethality (type II), the most severe form among surviving patients (type III), an intermediate form between type I and type III (type IV), and the unique type with interosseous membrane calcification of the forearm and/or hypertrophic callus (type V) (3, 4).

Due to the compromised mechanical strength of bone and low bone mass with high material density (5), patients with OI have a high risk of fractures throughout their lifetime (6, 7). The fracture incidence is highest during childhood and then declines in midlife, while remaining higher than that of the general population (8). Further, the fracture risk increases again in the elderly age group (9). In postmenopausal women and men over 55 years old with OI, the fracture rates are 4.9- to 8.0-fold higher than those in non-OI individuals (9). Therefore, the higher fracture risk in adult patients with OI still requires attention and effective medical therapy.

The current management of OI is focused on reducing fracture incidence (10). Bisphosphonates (BPs), which can bind to hydroxyapatite and exert their antiresorptive action on osteoclasts, have been extensively used in children with OI (11). Studies have indicated that BPs can decrease bone resorption, increase bone mineral density (BMD), and reduce the fracture risk of adult patients with OI (8, 12). However, BPs have been shown to be less effective in reducing bone fracture risk in patients with severe OI (13, 14). Therefore, new therapeutic agents urgently need to be explored to treat OI. Recently, denosumab, a monoclonal antibody targeting RANKL, was approved for the treatment of postmenopausal osteoporosis and male osteoporosis, which can inhibit the activities of osteoclasts, increase BMD, and reduce fracture risk (15). Small sample studies have evaluated the effects of denosumab in OI children with a poor response to BPs, but the efficacy and safety of denosumab in adult patients with OI are unclear (16, 17). One clinical trial evaluating efficacy of denosumab in children with OI was terminated because of frequent side effects with hypercalcemia (NCT02352753) (18). Head to head clinical studies comparing the efficacy of denosumab vs BPs have not yet been reported in adult patients with OI.

Therefore, we evaluated the safety and efficacy of denosumab in adult patients with OI by comparing it head to head with zoledronic acid.

## Materials and Methods

### Study Design

This was a 12-month, prospective, randomized, open-label study. Adult patients with OI were randomized to receive either subcutaneous injections of 60 mg of denosumab (Prolia, Amgen Inc., USA) every 6 months or 1 intravenous infusion of 5 mg of zoledronic acid (Aclasta, Novartis Pharmaceuticals, Switzerland). All patients were supplemented with 600 mg of calcium and 0.25 μg of calcitriol daily.

The study was approved by the Scientific Ethics Committee of Peking Union Medical College Hospital (PUMCH, JS-3545D) and was conducted in accordance with the declaration of Helsinki. All patients with OI provided written informed consent before they participated in this study.

### Subjects

Patients aged 18 years or older with clinically diagnosed OI were eligible. A clinical diagnosis of OI was made when the patient met 1 of the following criteria: (1) a history of at least 1 fracture under minor trauma during childhood and an age- and gender-adjusted BMD Z-score less than −2.0 at lumbar spine (LS) or proximal femur before any antiosteoporosis therapy; (2) presence of blue sclera or dentinogenesis imperfecta and a family history of OI (19). We excluded patients with an estimated glomerular filtration rate lower than 35 mL/minute, hypocalcemia, other disease history that could affect bone metabolism, ongoing treatment with glucocorticoids, cancer, or allergy to denosumab or zoledronic acid. Patients discontinuing previous osteoporosis medications at the screening visit are eligible for inclusion, except for those who received intravenous zoledronic acid within the 12 months before screening.

### Study Assessments

Genomic DNA of patients with OI was extracted from peripheral leukocytes by a standard procedure (DNA Extraction Mini Kit, Qiagen, Frankfurt, Germany). Next-generation sequencing (NGS) was completed to identify the pathogenic variants under the protocol (Illumina HiSeq2000 platform, Illumina Inc., San Diego, CA, USA) (19). The NGS panel covers more than 800 candidate genes of disorders related to bone, including 20 known candidate genes for OI (*COL1A1*, *COL1A2*, *IFITM5*, *SERPINF1*, *CRTAP*, *P3H1*, *PPIB*, *SERPINH1*, *FKBP10*, *PLOD2*, *BMP1*, *SP7*, *TMEM38B*, *WNT1*, *CREB3L1, SPARC*, *MBTPS2*, *P4HB*, *SEC24D*, and *PLS3*). Polymerase chain reaction was performed using the designed primers by a 3730 DNA analyzer (Applied Biosystems, Foster City, CA, USA), and Sanger sequencing was completed to confirm the mutations identified by NGS. According to the inherited mode, patients with OI were divided into autosomal dominant (AD) and non-AD groups. The AD group included patients with mutations in *COL1A1*, *COL1A2*, *IFITM5*, or *P4HB*, and patients carrying the other gene mutations were classified as the non-AD group. As for *COL1A1* and *COL1A2* variants, based on their impact on the synthesis of type I collagen, mutations leading to amino acid substitutions in the triple helical domain of *COL1A1* or *COL1A2* were categorized as collagen qualitative defects, while nonsense or frameshift mutations causing a premature stop codon in *COL1A1* were classified as collagen quantitative reduction (13). Other mutations, such as splicing mutations, were not evaluated here due to the difficulty in predicting their effects.

Areal BMD (aBMD) at the LS, femoral neck (FN), and total hip (TH) was measured at 0, 6, and 12 months of treatment using dual-energy X-ray absorptiometry (DXA, Lunar Prodigy Advance, GE Healthcare, USA). A quality control program was conducted throughout the study, and the phantom was tested daily using the DXA device for calibration and quality checks. All scans of patients were performed on the same densitometer. Obviously compressed or deformed vertebrae were excluded from the BMD analysis. The coefficients of variation of BMD at LS, FN, and TH were 1.0%, 1.6%, and 0.7%, and the least significant changes were 2.2%, 3.9%, and 2.2%, respectively (20).

The trabecular bone score (TBS) was extracted from the DXA image using TBS iNsight software (version 2.1; Medimaps, Merignac, France). LS TBS was calculated as the mean value of the measurement of L1 to L4, and vertebral compression fracture (VCF) or evident deformities were excluded from the calculation of LS TBS. The precision for TBS was 1.14% (21).

Fasting serum was collected at 8:00 to 10:00 Am at baseline and after 6 and 12 months of treatment. Serum levels of β-C-terminal telopeptide of type 1 collagen (β-CTX, a bone resorption marker), 25-hydroxyvitamin D (25OHD), and intact parathyroid hormone (PTH) were measured using an automated electrochemiluminescence system (E170, Roche Diagnostics, Switzerland). Serum levels of alkaline phosphatase (ALP, a bone formation marker), alanine aminotransferase (ALT), creatinine, albumin-adjusted calcium (Ca), and phosphate (P) were measured using automated analyzers (ADVIA 1800, Siemens, Germany). All parameters were detected in the central clinical laboratory of PUMCH.

Previous fracture history refers to all fragility fractures that occurred before patients with OI enrolled in this study. Previous fracture history was obtained from self-report by the patients, including the age, the cause (high trauma or minimal trauma), and the site of fracture. VCF was evaluated by anteroposterior and lateral radiographs of the thoracic and lumbar spine at baseline and at the end of the study, which was diagnosed according to the Genant semiquantitative method (22). New nonvertebral fractures were initially self-reported by the patients and then confirmed by X-ray films.

### Study Endpoints

The primary efficacy endpoints were the changes in aBMD at the LS, FN, and TH from baseline to 6 and 12 months of treatment. Secondary endpoints were the changes in the TBS, serum β-CTX, and ALP levels from baseline to 6 and 12 months of treatment. Additional end points included the incidence of new fractures and changes in serum levels of Ca, P, PTH, and 25OHD during the treatment. The changes in aBMD, TBS, and bone turnover biomarkers (BTMs) were evaluated in subgroups of patients with different inheritance patterns (AD or non-AD) and abnormalities of type I collagen (quantitative reduction or qualitative defect).

Safety of the treatment was assessed by clinical records and laboratory tests. All participants were questioned concerning adverse events (AEs) at each visit.

### Statistical Analysis

The Kolmogorov–Smirnov test was performed to test the normal distribution of continuous variables. Normally distributed data (including aBMD, TBS, ALP, β-CTX, PTH, Ca, P, and 25OHD at baseline and at each visit, previous fracture incidence, etc.) are presented as the mean ± SD. Within-group comparisons of changes in aBMD, TBS, and BTMs from baseline to each visit were conducted using paired t tests. Independent t tests were utilized to compare the differences in baseline data and percentage changes in the TBS and BTMs between the 2 groups. Non-normally distributed variables (percentage change in aBMD, previous treatment duration, and serum ALT) were expressed as medians and interquartile ranges (IQRs), and differences in the percentage change in aBMD between the 2 groups were compared with the Mann–Whitney U test. Qualitative data, such as previous treatment history and previous VCF, are expressed as numbers and proportions, which were analyzed by Fisher’s exact test between the 2 groups.

Percentage changes in aBMD, TBS, and BTMs from baseline to 12 months in subgroups of patients with different inheritance patterns (AD or non-AD), abnormalities of type I collagen (quantitative reduction or qualitative defect) and Sillence type (type I or type III/IV) were assessed by the independent t test.

The primary efficacy analysis was conducted on the basis of the full analysis set, which included all patients who were randomly assigned and received at least 1 dose of medication. All baseline demographic data were also analyzed on the basis of the full analysis set. Safety analysis included all patients who underwent randomization and received at least 1 dose of the therapeutic drugs. Safety endpoints included the incidence and severity of AEs, changes in vital signs from baseline and laboratory values, and the incidence of new fractures. Fisher’s exact test was used to compare the incidence of AEs between the 2 groups.

Statistical analyses were performed using SPSS software (version 26.0; SPSS Inc., Chicago, IL, USA). Statistical significance was considered when the 2-tailed *P* < .05.

## Results

### Baseline Characteristics

**Figure 1.**
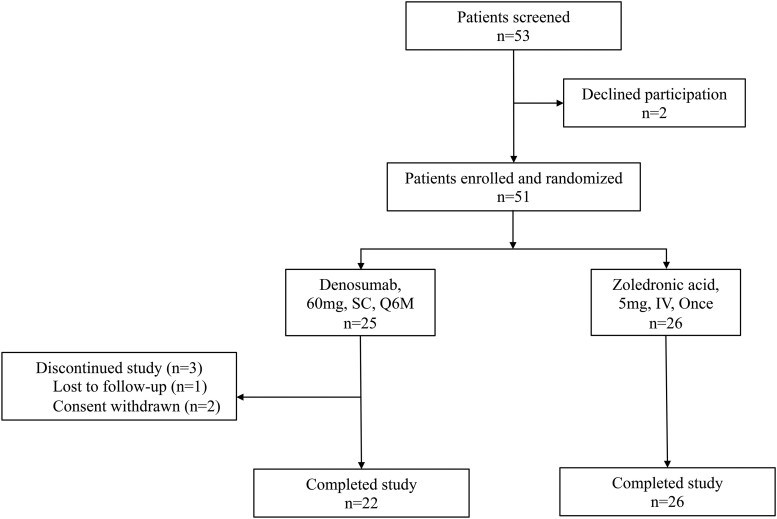
Flow chart of the study. IV, intravenous transfusion; OI, osteogenesis imperfecta; Q6M, every 6 months; SC, subcutaneous injection.

A total of 51 adult patients with OI were enrolled into the study, and 48 (94.1%) patients completed 12 months of follow-up (denosumab: 88.0%, zoledronic acid: 100.0%) (Fig. 1). Three patients in the denosumab group terminated the study early: 1 patient was lost to follow-up because of relocation and the other 2 patients refused regular follow-up.

At baseline, the demographics and clinical characteristics were similar between the 2 treatment groups (Table 1). The mean age of the patients in the denosumab and zoledronic acid groups were 36.7 ± 12.7 and 37.1 ± 11.8 years, respectively. The previous fracture incidences were 0.28 ± 0.30 and 0.31 ± 0.27 per year in the denosumab and zoledronic acid groups, respectively. VCF were found in 52.0% and 61.5% of patients in the denosumab and zoledronic acid group (*P* = .492). Pain was reported in 24.0% (6/25) and 26.9% (7/26) of the denosumab and zoledronic acid groups (*P* = .811), while ambulation with aids was reported in 36.0% (9/25) and 46.2% (12/26) (*P* = .461), respectively. Based on the Sillence classification, the distribution of OI severity was similar in the 2 groups. The mean aBMD at the LS, FN, and TH, TBS, and the serum levels of β-CTX, ALP, Ca, P, PTH, and 25OHD also showed no significant differences between the 2 groups at baseline (Table 1).

**Table 1.** Baseline demographic and clinical characteristics of the patients with OI

	Denosumab (n = 25)	Zoledronic acid (n = 26)	*P* value	Reference range
Age (±SD), year	36.7 ± 12.7	37.1 ± 11.8	.909	
Female/Male, n	14/11	14/12	.877	
Height (±SD), m	1.53 ± 0.15	1.51 ± 0.18	.675	
Weight (±SD), kg	57.92 ± 12.30	56.38 ± 12.31	.658	
BMI (±SD), kg/m^2^	24.70 ± 4.17	25.02 ± 5.48	.816	
Sillence type, I/III/IV, n	12/7/6	14/8/4	.812	
Pain, n (%)	6 (24.0%)	7 (26.9%)	.811	
Ambulation with aids*^a^*, n (%)	9 (36.0%)	12 (46.2%)	.461	
Previous fracture incidence (±SD), n/year/person	0.28 ± 0.30	0.30 ± 0.28	.759	
Previous VCF, n (%)	13 (52.0%)	16 (61.5%)	.492	
Previous treatment history, n (%)	17 (68.0%)	16 (61.5%)	.629	
Alendronate	9 (36.0%)	11 (42.3%)	.645	
Zoledronic acid	9 (36.0%)	8 (30.8%)	.771	
Teriparatide	3 (12.0%)	1 (3.8%)	.350	
Previous treatment duration (IQR), year	1.00 (0.00-4.00)	0.75 (0.00-2.19)	.345	
ALT (IQR), U/L	17.00 (12.50-25.50)	19.00 (14.75-34.50)	.257	7-40
Cr (±SD), μmol/L	58.04 ± 14.05	58.08 ± 11.23	.992	45-84
Ca (±SD), mmol/L	2.39 ± 0.08	2.37 ± 0.08	.588	2.13-2.70
P (±SD), mmol/L	1.16 ± 0.14	1.06 ± 0.19	.180	0.81-1.45
25OHD (±SD), ng/mL	25.38 ± 9.86	19.38 ± 7.39	.190	>30
PTH (±SD), pg/mL	41.98 ± 13.51	47.08 ± 25.23	.535	15.0-65.0
ALP (±SD), U/L	85.17 ± 37.39	88.12 ± 36.76	.782	35-100
β-CTX (±SD), ng/mL	0.27 ± 0.22	0.26 ± 0.22	.858	0.21-0.44
LS, g/cm^2^	0.84 ± 0.22	0.84 ± 0.13	.963	0.97-1.05 (23)
LS Z-score	−2.17 ± 1.77	−1.89 ± 1.15	.573	> −2.0
FN, g/cm^2^	0.70 ± 0.18	0.78 ± 0.15	.148	0.78-1.00 (24)
FN Z-score	−1.67 ± 1.47	−0.98 ± 1.19	.109	> −2.0
TH, g/cm^2^	0.72 ± 0.17	0.78 ± 0.15	.225	—
TH Z-score	−1.76 ± 1.26	−1.29 ± 1.11	.210	> −2.0
TBS	1.29 ± 0.13	1.27 ± 0.18	.604	≥1.35

Data are presented as the median (IQR), number (%), or mean (SD).

Bold numbers represent *P* < .05.

Abbreviations: 25OHD, 25-hydroxyvitamin D; β-CTX, β-C-terminal telopeptide of type 1 collagen; ALP, alkaline phosphatase; ALT, glutamic-pyruvic transaminase; BMD, bone mineral density; BMI, body mass index; Ca, albumin-adjusted calcium; Cr, creatinine; FN, femoral neck; LS, lumbar spine; OI, osteogenesis imperfecta; P, phosphorus; PTH, parathyroid hormone; TH, total hip; VCF, vertebral compression fracture.

^*a*^Defined as difficulty in walking independently, requiring dependence on tools such as wheelchairs, walkers, crutches, etc.

**Figure 2.**
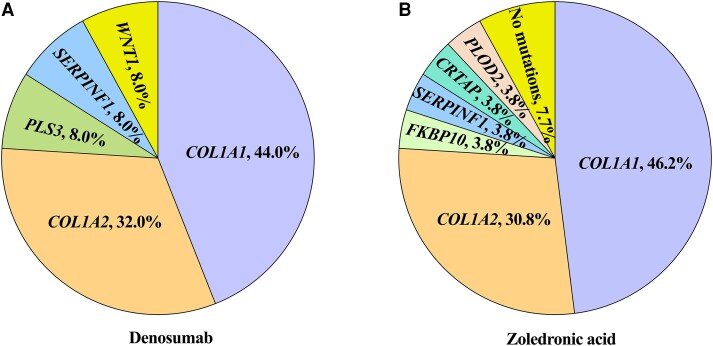
Gene mutation spectrum of patients with OI. (A) Gene mutation spectrum in the denosumab group. (B) Gene mutation spectrum in the zoledronic acid group. OI, osteogenesis imperfecta.

Pathogenic mutations were identified in 49 patients with OI, with 25 in the denosumab group and 24 in the zoledronic acid group (Fig. 2). A total of 8 OI-related genes were identified, and mutations in *COL1A*1 (denosumab: 11/25, 44.0%; zoledronic acid: 12/26, 46.2%) and *COL1A2* (denosumab: 8/25, 32.0%; zoledronic acid: 8/26, 30.8%) were the most common, which were classified as AD inheritance. Mutations in *WNT1*, *SERPINF1*, and *PLS3* were identified in the denosumab group, with a mutational frequency of 2/25 (8.0%) for each gene. Mutations in *SERPINF1*, *FKBP10*, *CRTAP*, and *PLOD2* were identified in the zoledronic acid group, with a mutational frequency of 1/26 (3.8%) for each gene. Pathogenic mutations in these genes were categorized as non-AD inheritance. The proportion of patients with AD or non-AD inheritance did not differ between the 2 groups.

### Efficacy of Denosumab or Zoledronic Acid Treatment

#### Bone mineral density

**Figure 3.**
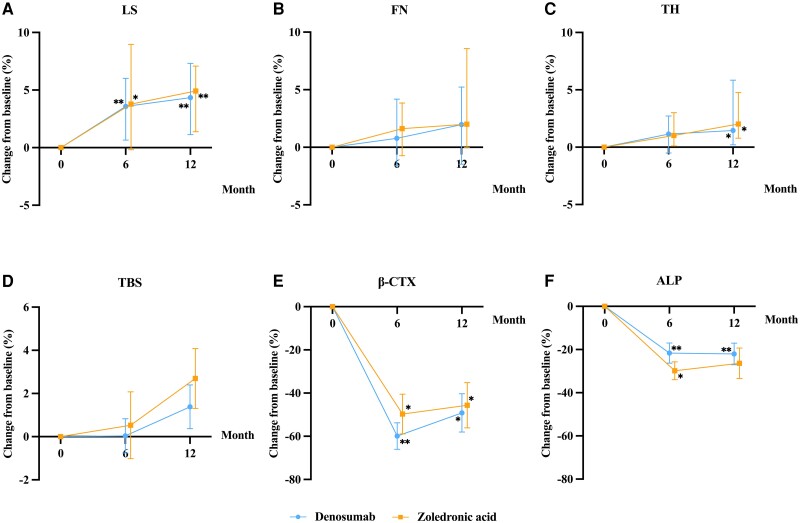
Percentage changes in aBMD, TBS, and BTMs after denosumab or zoledronic acid treatment. (A) Median percentage changes in aBMD at the LS during treatment. (B) Median percentage changes in aBMD at the FN during treatment. (C) Median percentage changes in aBMD at the TH during treatment. (D) Median percentage changes in the TBS during treatment. (E) Mean percentage changes in the serum levels of β-CTX during treatment. (F) Mean percentage changes in the serum levels of ALP during treatment. β-CTX, β-C-terminal telopeptide of type 1 collagen; aBMD, areal bone mineral density; ALP, alkaline phosphatase; BTM, bone turnover marker; FN, femoral neck; LS, lumbar spine; TBS, trabecular bone score; TH, total hip. Data are shown as the mean and standard error or median (IQR). *P < .05, **P < .01 vs baseline.

At 6 and 12 months of treatment, denosumab significantly increased aBMD at the LS by 3.59% (*P* = .004) and 4.34% (*P* = .005) from baseline, and zoledronic acid also resulted in a significant increase in LS aBMD by 3.78% (*P* = .011) and 4.92% (*P* = .006) from baseline (Fig. 3A).

At 12 months of treatment, aBMD at the TH showed a notable increase of 1.45% (*P* = .023) and 2.02% (*P* = .016) from baseline in the denosumab and zoledronic acid groups, respectively, whereas no significant change was found in aBMD at the TH at 6 months of denosumab or zoledronic acid treatment (Fig. 3C). Additionally, no significant increase in aBMD at the FN was found at 6 or 12 months of denosumab or zoledronic acid treatment (Fig. 3B).

Furthermore, no significant differences in the aBMD changes at the LS, FN, and TH were found between the denosumab and zoledronic acid groups during the whole treatment period (all *P* > .05) (Fig. 3A-3C).

#### Trabecular bone score

After 6 months of treatment, we observed a slight increase in the TBS in the denosumab (0.04 ± 2.97%) and zoledronic acid (0.53 ± 5.99%) groups, and the trend of increase was more noticeable at 12 months of denosumab (1.39 ± 3.80%) and zoledronic acid (2.70 ± 5.57%) treatment, although not reaching statistical significance. Additionally, the percentage changes in the TBS were comparable between the 2 groups (*P* > .05) (Fig. 3D).

#### Serum levels of serum biochemical indexes

The mean level of serum β-CTX significantly declined by 49.71 ± 9.20% and 45.64 ± 10.45% after 6 and 12 months of denosumab treatment, and by 59.88 ± 24.61% and 49.18 ± 35.51% after 6 and 12 months of zoledronic acid treatment (all *P* < .05 vs baseline). No statistically significant differences were observed with respect to changes in β-CTX and ALP levels between the 2 groups during the whole treatment (Fig. 3E and 3F).

The mean serum PTH level reduced by 16.45% after 12 months of denosumab treatment (*P* = .015 vs baseline), while remaining within the normal reference range (Fig. S1C (25)). Other serum biochemical markers, such as Ca, P, and 25OHD, showed no significant changes from baseline (Fig. S1 (25)), with no notable differences observed between the denosumab and zoledronic acid groups.

#### New fracture incidence

During the 12-month period of treatment, 3 patients experienced new fractures: 2 (8.0%) in the denosumab group (right femur and right ulna) and 1 (3.8%) in the zoledronic acid group (right femoral shaft). The incidence of new fractures showed no significant difference between the 2 groups (Table 2).

**Table 2.** AEs during denosumab and zoledronic acid treatment

	Denosumab (n = 25)	Zoledronic acid (n = 26)	*P* value
Injection site reaction, n (%)	1 (4.0)	2 (7.7)	1.000
New fracture, n (%)	2 (8.0)	1 (3.8)	.610
Acute-phase reaction, n (%)	0 (0.0)	9 (34.6)	**.002**
Pyrexia	0 (0.0)	7 (26.9)	**.010**
Myalgia	0 (0.0)	2 (7.7)	.490
Headache	0 (0.0)	0 (0.0)	—
Other events of interest, n (%)			
Hypercalcemia	0 (0.0)	0 (0.0)	—
Hypocalcemia	0 (0.0)	0 (0.0)	—
Bone pain	3 (12.0)	5 (19.2)	.703
Fatigue	1 (4.0)	0 (0.0)	.490
Osteonecrosis of the jaw	0 (0.0)	0 (0.0)	—
Atypical fracture	0 (0.0)	0 (0.0)	—
Soft tissue infections	0 (0.0)	0 (0.0)	—
Serious AEs, n (%)	0 (0.0)	0 (0.0)	—
All AEs, n (%)	5 (20.0)	14 (53.8)	**.012**

Bold numbers represent *P* < .05.

Abbreviations: AEs, adverse events.

### Correlation of genotype or Sillence type and response to denosumab and zoledronic acid treatment

**Figure 4.**
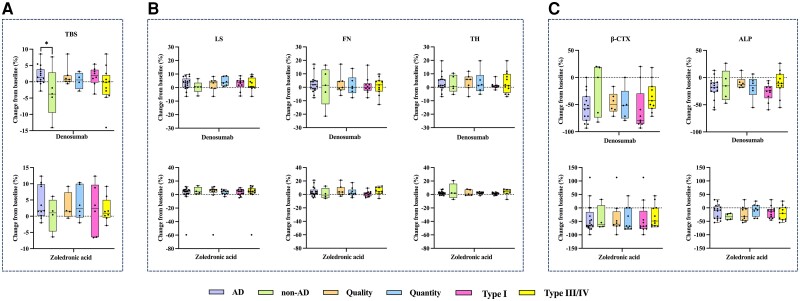
Correlation of pathogenic mutation or Sillence type and response to denosumab and zoledronic acid treatment. (A) Percentage changes in the TBS in patients with AD and non-AD inheritance, qualitative defects, and quantitative reductions in type I collagen, and type I and type III/IV after 12 months of denosumab and zoledronic acid treatment. (B) Percentage changes in aBMD at the LS, FN, and TH in patients with AD and non-AD inheritance, qualitative defects and quantitative reductions in type I collagen, and type I and type III/IV after 12 months of denosumab and zoledronic acid treatment. (C) Percentage changes in serum levels of β-CTX and ALP in patients with AD and non-AD inheritance, qualitative defects, and quantitative reductions in type I collagen, and type I and type III/IV after 12 months of denosumab and zoledronic acid treatment. β-CTX, β-C-terminal telopeptide of type 1 collagen; AD, autosomal dominant (inheritance); ALP, alkaline phosphatase; BMD, bone mineral density; FN, femoral neck; LS, lumbar spine; non-AD, autosomal recessive or X-linked (inheritance); OI, osteogenesis imperfecta; Quantity, quantitative reduction in type I collagen; Quality, qualitative defect of type I collagen; TBS, trabecular bone score; TH, total hip. *P < .05 AD vs non-AD.

Figure 4 shows the percentage changes in aBMD, TBS, and BTMs in patients with OI with different inheritance patterns (AD, denosumab: n = 17, zoledronic acid: n = 20; non-AD, denosumab: n = 5; zoledronic acid: n = 4), abnormalities of type I collagen (quantitative reduction, denosumab: n = 7, zoledronic acid: n = 10; qualitative defect, denosumab: n = 6, zoledronic acid: n = 9), and Sillence type (type I, denosumab: n = 10, zoledronic acid: n = 14; type III/IV, denosumab: n = 12, zoledronic acid: n = 12). After 12 months of denosumab treatment, patients with AD inheritance showed a significantly higher percentage increase in the TBS than those with non-AD inheritance (1.98 ± 2.87% vs −3.41 ± 7.76%, *P* = .030) (Fig. 4A). Additionally, patients with OI with AD inheritance showed a trend toward higher percentage increases in aBMD at the LS (3.18 ± 4.38% vs 0.29 ± 5.19%), FN (2.25 ± 6.31% vs 0.56 ± 14.49%), and TH (3.15 ± 6.31% vs 2.59 ± 6.46%), along with a trend toward a greater decrease in β-CTX levels (−54.87 ± 27.56% vs −21.86 ± 48.38%) than those with non-AD inheritance, although this did not reach statistical significance. Similarly, patients with a quantitative reduction in type I collagen exhibited a trend toward a higher increase in LS aBMD (4.51 ± 3.32% vs 2.04 ± 4.84%) and a greater reduction in the ALP level (−17.96 ± 21.04% vs −9.38 ± 11.77%) than those with a qualitative defect of type I collagen. Patients with OI type I showed a trend toward a higher increase in TBS (1.60 ± 2.86% vs 0.24 ± 3.98%) and greater reductions in serum ALP (−26.94 ± 16.34% vs −14.70 ± 20.67%) and β-CTX levels (−59.73 ± 41.01% vs −35.89 ± 27.95%) than those with OI type III/IV.

Similarly, after 12 months of zoledronic acid treatment, patients with OI with AD inheritance showed a trend toward higher increases in FN aBMD (4.09 ± 7.00% vs 1.14 ± 8.06%) and TBS (4.30 ± 5.11% vs 0.45 ± 5.18%) than those with non-AD inheritance. Furthermore, patients with OI type I presented higher percentage increases in LS aBMD (3.65 ± 4.34% vs 0.75 ± 19.39%) and TBS (2.18 ± 7.74% vs 1.94 ± 3.88%) than those with OI type III/IV.

The treatment response of each patient with non-AD inheritance to denosumab and zoledronic acid are presented elsewhere (Fig. S2 (25)). Overall, both groups demonstrated mean percentage increases in BMD after 12 months of treatment; nevertheless, notable heterogeneity in BMD change was observed among different individuals.

### Safety

The overall incidence of AEs was significantly lower in the denosumab group than in the zoledronic acid group (20.0% vs 53.8%, *P* = .012), primarily attributed to a notably lower occurrence of acute-phase reactions in the denosumab group in comparison to the zoledronic acid group (0.0% vs 34.6%, *P* = .002). Other AEs included bone pain (denosumab: n = 3, zoledronic acid: n = 5), injection site reaction (denosumab: n = 1, zoledronic acid: n = 2), and fatigue (denosumab: n = 1, zoledronic acid: n = 0), which were all mild and did not lead to discontinuation of treatment. During the entire study period, no osteonecrosis of the jaw or atypical fracture was found, and no serious AEs were observed (Table 2).

## Discussion

Currently, pharmacotherapy studies are extremely rare in adult patients with OI. In this study, we prospectively evaluated the efficacy and safety of denosumab by comparing it head to head with zoledronic acid for the first time in a cohort of adult patients with OI. Our results showed that treatment with denosumab for 1 year significantly increased aBMD at the LS and TH and markedly reduced bone resorption in adult patients with OI. The efficacy of denosumab was similar to that of zoledronic acid, but denosumab treatment resulted in fewer AEs than zoledronic acid therapy during the study period. Additionally, our findings indicated that patients with OI with AD inheritance may have a better response to denosumab treatment, as demonstrated by a significantly higher percentage increase in the TBS at 12 months.

Although patients with OI have increased skeletal fragility and a high fracture risk throughout their lifetimes, treatment is often overlooked in adult patients with OI, as the fracture risk usually declines in middle age. Therapeutic studies are scarce in adult patients with OI (8). Recently, several studies, including 7 prospective studies and 1 retrospective case series, reported the outcomes of BP treatment in adult patients with OI (8). BPs have been shown to be beneficial in improving BMD and reducing fracture risk in adult patients with OI to some extent (26-28). Since OI is a genetically and clinically heterogeneous disease, the outcome of BP treatment varies with the severity of OI. Our previous prospective study revealed that OI children with a more severe phenotype had a poorer response to zoledronic acid treatment than those with a milder phenotype (13), and similar results were also found in an observational study (29). Moreover, BPs can stay in the skeleton for several years, raising concerns about their long-term side effects. Therefore, new agents are worth investigating in patients with OI, especially in adult patients with OI.

Recently, denosumab has been shown to be able to reduce bone resorption, increase BMD and reduce fracture risk through targeting the bone resorption mediator RANKL (30). However, the efficacy of denosumab is unclear in patients with OI. A few small-scale studies involving OI children indicated that treatment with denosumab significantly improves BMD and longitudinal growth (16, 31-33). One prospective clinical trial in children with OI treated with denosumab was stopped because of rebound hypercalcemia (18). In 1 retrospective study, 5 adult patients with OI received treatment with denosumab (16). Except for 1 patient, increases in BMD at the LS and TH were observed, and no fractures occurred during a median treatment period of 18 months (16). In our study, we found that denosumab treatment for 12 months significantly increased aBMD at the LS and TH of adult patients with OI, which would provide new evidence for the clinical application of denosumab in adult patients with OI. Two head to head studies have compared the effects of denosumab and zoledronic acid in postmenopausal women with osteoporosis; the results indicate that denosumab is superior or similar to zoledronic acid in increasing BMD and suppressing bone resorption (34, 35). In this head to head comparative study, we found that denosumab had the similar efficacy in improving BMD and decreasing BTMs to zoledronic acid in adult patients with OI.

Interestingly, the effects of denosumab therapy on the TBS were observed in this study. The TBS, a gray-level texture index derived from LS DXA scans, correlates with the bone microstructure and is valuable in fracture risk prediction in patients with osteoporosis (36). Recently, numerous studies have reported the effects of denosumab and zoledronic acid on TBS in patients with osteoporosis, but the results are inconsistent (37-40). Thus far, 3 studies have revealed a significant decline in the TBS in patients with OI (41-43). One study evaluated the effects of denosumab and zoledronic acid on the TBS in pediatric patients with OI, indicating that denosumab significantly increased the TBS, while zoledronic acid had no significant effects on the TBS (42). In patients with osteoporosis, TBS combined with BMD, is useful for evaluating treatment response to long-term denosumab (39, 44). However, the correlation between changes in TBS and fracture risk of patients with OI has not been explored. In this study, we did not observe a significant correlation between denosumab or zoledronic acid treatment and TBS improvement, although a trend of TBS increase was noted. Indeed, an increase in TBS might not be anticipated in patients with antiresorptive treatment, such as bisphosphonates (36). Additionally, the expert Working Group recommends TBS for evaluating the long-term denosumab treatment response (5 years or more) (36). Therefore, a longer follow-up is necessary to clarify the role of the TBS in evaluating denosumab efficacy in OI adults. On the other hand, we observed a more substantial increase in TBS among patients with OI with AD inheritance compared with those with non-AD inheritance, with no significant difference in BMD improvement between the 2 groups. These findings suggest that the TBS might serve as a sensitive indicator for precise therapy assessment in patients with OI, and it is also worth further investigation.

Denosumab was well-tolerated with fewer overall AEs than zoledronic acid during the study period. Previous studies have reported rebound hypercalcemia in children with OI after 7 to 20 weeks of denosumab treatment (45, 46), which may be attributed to their active bone remodeling state. Although relatively rare, adults can also experience rebound hypercalcemia after discontinuing denosumab (47). Notably, we did not observe hypercalcemia during the 12-month study period. However, at 12 months, serum calcium levels displayed an upward trend, while PTH exhibited a significant decrease in denosumab group. Previous studies have suggested that longer treatment durations are associated with a higher hypercalcemia incidence after denosumab discontinuation (48). Due to the relatively short follow-up period and the fact that both serum PTH and calcium values were within the normal range in this cohort, it is uncertain whether the decline in PTH is a precursor to the occurrence of hypercalcemia. Additionally, denosumab discontinuation often leads to a rapid increase in bone resorption biomarkers and a decline in BMD (49). Therefore, appropriate time to discontinue denosumab and optional sequential therapy following denosumab discontinuation in OI adults are worthy further investigation.

Additionally, the notable decrease in PTH observed in denosumab treatment group might also be associated with the correction of secondary hyperparathyroidism. Denosumab-induced hypocalcemia triggers PTH release, stimulating calcium release from bones, thereby raising serum calcium levels. The elevated serum calcium, in turn, activates negative feedback mechanisms, inhibiting further PTH release and reducing PTH levels. However, a previous study indicated that the overall risk of hypocalcemia during denosumab treatment was low (50). Moreover, adequate supplementation of calcium and vitamin D could be associated with a lower risk of hypocalcemia during denosumab treatment (51). In this cohort, all patients received daily supplementation with 600 mg of calcium and 0.25 μg of calcitriol. Therefore, the likelihood of hypocalcemia caused by denosumab was considered to be low.

Of note, 2 patients with no detectable OI-related gene mutations were included in this study. Patient 1, a 62-year-old female with a height Z-score of −1.45, encountered an unspecified fracture at age 3. During premenopausal periods at 35 and 48 years, she suffered mild-trauma fractures of the right ulna and ankle. A physical examination revealed blue sclera and spinal curvature. BMD T- and Z-scores were below −2.5 and −2.0, respectively. Patient 2, a 43-year-old female with a height Z-score of −1.23, has had blue sclera since birth and experienced childhood joint dislocations. At age 21, she sustained a pelvic fracture after a fall on the flat surface. A physical examination revealed blue sclera and joint ligament laxity. Additionally, this patient’s daughter was diagnosed with OI. The FN Z-score was −2.1 in this patient. Based on their clinical features, both patients were clinically diagnosed as OI. Indeed, 4 new OI pathogenic genes (*KDELR2*, *FAM46A*, *MESD*, and *CCDC134*) were not included in the NGS panel of this study, which might lead to a negative genetic test result of these 2 patients.

This is the first head to head comparative study of denosumab vs zoledronic acid in adult patients with OI. Our findings may provide a novel and meaningful basis of denosumab treatment for adult patients with OI. We not only used the TBS for the first time to assess the efficacy of denosumab and zoledronic acid in adult patients with OI but also found that the TBS may serve as a novel indicator for precise therapy evaluation in OI. During the treatment, all biochemical parameters were consistently tested in a single laboratory, and the BMD was measured using the same DXA machine, thus minimizing measurement bias. However, there were several limitations. No placebo control was set in this study. Some patients with OI had a previous history of antiosteoporosis therapy, which may have impact on the efficacy assessment of this study. Moreover, the sample size was relatively small, and the treatment duration was only 1 year, which makes it difficult to evaluate the impact of denosumab treatment on the incidence of new fractures.

In conclusion, the treatment of adults with OI should be considered if they have a low BMD or high fracture risk. In this head to head study, we demonstrated that denosumab treatment significantly reduced bone loss and increased the BMD in adult patients with OI, with comparable efficacy to zoledronic acid but fewer side effects. Further long-term and large-sample studies are still needed to confirm the antifracture efficacy and safety of denosumab in adult patients with OI.

## Data Availability

The data that support the findings of this study are available from the corresponding author upon reasonable request.

## Abbreviations

25OHD: 25-hydroxyvitamin D
β-CTX: β-C-terminal telopeptide of type 1 collagen
aBMD: areal bone mineral density
AD: autosomal dominant
AE: adverse event
ALP: alkaline phosphatase
ALT: alanine aminotransferase
BP: bisphosphonate
BTM: bone turnover biomarker
Ca: albumin-adjusted calcium
DXA: dual-energy X-ray absorptiometry
FN: femoral neck
IQR: interquartile range
LS: lumbar spine
NGS: next-generation sequencing
OI: osteogenesis imperfecta
P: phosphate
PTH: parathyroid hormone
TBS: trabecular bone score
TH: total hip
VCF: vertebral compression fracture
